# A novel immunofluorescent assay to investigate oxidative phosphorylation deficiency in mitochondrial myopathy: understanding mechanisms and improving diagnosis

**DOI:** 10.1038/srep15037

**Published:** 2015-10-15

**Authors:** Mariana C. Rocha, John P. Grady, Anne Grünewald, Amy Vincent, Philip F. Dobson, Robert W. Taylor, Doug M. Turnbull, Karolina A. Rygiel

**Affiliations:** 1 Newcastle University Centre for Ageing and Vitality, Institute for Neuroscience, Medical School, Newcastle University, Newcastle upon Tyne, United Kingdom; 2 Wellcome Trust Centre for Mitochondrial Research, Institute for Neuroscience, Medical School, Newcastle University, Newcastle upon Tyne, United Kingdom

## Abstract

Oxidative phosphorylation defects in human tissues are often challenging to quantify due to a mosaic pattern of deficiency. Biochemical assays are difficult to interpret due to the varying enzyme deficiency levels found in individual cells. Histochemical analysis allows semi-quantitative assessment of complex II and complex IV activities, but there is no validated histochemical assay to assess complex I activity which is frequently affected in mitochondrial pathology. To help improve the diagnosis of mitochondrial disease and to study the mechanisms underlying mitochondrial abnormalities in disease, we have developed a quadruple immunofluorescent technique enabling the quantification of key respiratory chain subunits of complexes I and IV, together with an indicator of mitochondrial mass and a cell membrane marker. This assay gives precise and objective quantification of protein abundance in large numbers of individual muscle fibres. By assessing muscle biopsies from subjects with a range of different mitochondrial genetic defects we have demonstrated that specific genotypes exhibit distinct biochemical signatures in muscle, providing evidence for the diagnostic use of the technique, as well as insight into the underlying molecular pathology. Stringent testing for reproducibility and sensitivity confirms the potential value of the technique for mechanistic studies of disease and in the evaluation of therapeutic approaches.

Defects of mitochondrial oxidative phosphorylation (OXPHOS) are found in a wide range of human pathologies, either as a primary cause of disease through genetic defects involving either the mitochondrial (mtDNA) or nuclear genome^1^,^2^, or secondary when there are other prominent pathological processes such as inflammation (multiple sclerosis^3^, inclusion body myositis^4^) or degenerative features (Parkinson’s disease^5^).

Skeletal muscle is frequently affected by both primary and secondary mitochondrial defects. Mitochondrial myopathies are progressive and clinical features include chronic progressive external ophthalmoplegia, rhabdomyolysis, muscle fatigue and severe proximal weakness^6^. Muscle is also commonly affected in patients with a multisystem phenotype^7^,^8^. Involvement of mtDNA usually leads to mosaic deficiency of the OXPHOS in muscle; mtDNA is present in multiple copies in cells and mutations are frequently heteroplasmic (mixed mutated and wild-type mtDNA within an individual cell). Biochemical defects are often only apparent in cells with high levels of mutated mtDNA^9^,^10^.

Current diagnostic algorithms for investigating and diagnosing mitochondrial disease use histochemical and biochemical assessment of OXPHOS activities in clinically-affected tissues^11^. Biochemical assays measure activity of each individual OXPHOS enzyme complex (complex I–V) in homogenised muscle tissue^11^. Whilst they are very useful for assessment of wide-spread mitochondrial defects, they may fail to detect more subtle OXPHOS deficiencies, especially when only a few myofibres are involved. They also require substantial quantities of muscle (>50 mg) which are not always available.

The histochemical assessment of cytochrome *c* oxidase (Complex IV, COX) and succinate dehydrogenase (Complex II, SDH) activities are standard methodologies to assess OXPHOS function in tissue cryosections, interrogating the activities of these two complexes in individual cells^12^,^13^. Though valuable for detecting the mosaic OXPHOS deficiency observed in many mitochondrial myopathies, myofibre classification is subjective, qualitative and prone to inter-observer variability^14^,^15^. Further, there are no histochemical techniques to assess other OXPHOS complexes, in particular complex I, which is commonly affected in mitochondrial disease^16^,^17^, and often occurs at initial stages of cell damage, a potentially valuable early indicator of pathology^18^.

As well as diagnostic applications, histochemical and biochemical assays have been used to investigate mitochondrial disease progression^19^,^20^ and to assess experimental therapeutic approaches in mitochondrial disease^21^,^22^. The effects of exercise and other potential therapies on mitochondrial function may be subtle, thus sensitive and quantitative methods are needed.

To overcome the limitations of currently available methods we have developed an objective and semi-automated technique allowing quantification of mitochondrial dysfunction in individual myofibres.

## Methods

### Cohort clinical characteristics

Muscle biopsies were obtained from twenty-two patients with clinically and genetically-characterised mitochondrial disease of both mtDNA and nuclear genetic origin, and from four healthy and disease controls (Table 1). All disease controls showed normal muscle histology, oxidative enzyme histochemistry, and OXPHOS biochemical activities^11^.

Ethical approval was granted by the Newcastle and North Tyneside Local Research Ethics Committees (reference 09/H0906/75). The experiments were carried out in accordance with the approved guidelines. Written informed consent was received from participants prior to inclusion in the study.

### COX/SDH histochemistry

Sequential COX/SDH histochemistry (COX/SDH) was carried out according to an established protocol^12^. Briefly, frozen muscle sections (10 μm) were dried, washed in PBS (OXOID, Basingstoke), and incubated with COX solution for 45 min at 37 °C. Following a PBS wash the sections were overlaid with SDH solution and incubated for 40 min at 37 °C.

### Quadruple immunofluorescence

Quadruple immunofluorescence was carried out on transverse muscle sections (10 μm) using antibodies detecting subunits of OXPHOS complexes (Supplementary Table S1). Complex I was detected using an antibody against subunit NDUFB8^23^, and Complex IV using an antibody to mtDNA encoded subunit I (COX-I). Mitochondrial mass was quantified using an antibody to porin, an outer mitochondrial membrane voltage-gated ion channel. Laminin, a basement membrane glycoprotein, was used to label the myofibre boundaries (Supplementary Table S1). Briefly, the sections were fixed in cold 4% paraformaldehyde (Sigma) for 3 min and permeabilised in a methanol (Fisher) gradient (10 min 70% methanol, 10 min 95% methanol, 20 min 100% methanol, 10 min 95%.methanol and 10 min 70% methanol). Non-specific protein interactions were blocked with 10% normal goat serum (Sigma) and incubated with the primary antibodies in a humidified chamber at 4 °C overnight (Supplementary Table S1). Following washes in TBST (Sigma), the sections were incubated with the secondary antibodies for 2 h at 4 °C and subsequently with streptavidin conjugated with Alexa 647 (Life Technologies) for 2 h at 4 °C (Supplementary Table S1). The sections were washed and mounted in Prolong Gold (Sigma). No-primary antibody controls, incubated only with anti-laminin antibody, were processed for each muscle sample.

### Image acquisition

Brightfield and fluorescent images were acquired at 20× magnification using a Zeiss Axio Imager M1 and Zen 2011 (blue edition) software, with a monochrome Digital Camera (AxioCam MRm) and filter cubes for Alexa Fluor dyes at 405 nm, 488 nm, 546 nm and 647 nm wavelengths, for laminin, COX-I, porin and NDUFB8 respectively. Exposure times for each channel were set to avoid pixel saturation and maintained between cases. Images were recorded as 16-bit czi files. Between 447 and 2400 fibres were imaged according to the muscle section size.

To assess reliability of immunofluorescence: fluorescent images were acquired at 20× magnification using a Zeiss Axio Imager Z2 with a motorized stage and AxioVision (Release.4.8.2) software, with identical camera and filters as above. Images were recorded as zvi files and processed by Zen 2011 (blue edition) software using the stitching function.

### Subjective assessment of myofibres (visual classification)

To assess the correlation between enzyme activity and protein abundance, visual classification of COX/SDH was performed using a stereological workstation with a modified light microscope (Olympus, Japan), motorized stage, CCD colour camera and stereology software (Stereo Investigator, MBF Bioscience, USA).

To assess the limitations of COX/SDH, a third investigator imaged different areas of two biopsies and selected 100 myofibres for each patient. Both investigators 1 and 2 visually classified labelled myofibres in COX/SDH images (at 20× magnification).

### Objective assessment of myofibres

#### Densitometry measurements

Fluorescent images were analysed using IMARIS software (Bitplane). Segmentation was performed to isolate individual myofibres (Supplementary Fig. S1). A first surface was created over the 405 nm channel and used to form a laminin mask. Following this, a second surface was created based on the masked 405 nm channel. As a result, multiple surfaces were formed over individual muscle fibres. Unwanted areas (such as vessels or background) were removed in a filtering step. The mean optical density (OD) of 488, 546 and 647 nm channels were automatically measured in each individual muscle fibre in a scale ranging from 0 (brightest pixels) to 65,535 (darkest pixels) intensity units (ODCOX-I, ODNDUFB8 and ODporin). The mean ODs of the no primary control (OD_488_, OD_546_, OD_647_) were measured to determine the levels of non-specific fluorescence.

#### Data analysis

For each myofibre, ODCOX-I, ODNDUFB8, and ODporin, were corrected for background signal. ODCOX-I and ODporin values were corrected by subtracting the mean OD_488_ and mean OD_546_ of the no primary control respectively. ODNDUFB8 was corrected according to mitochondrial mass: fibres were sorted into one hundred groups according to the ODporin percentile, and corrected using the mean OD_647_ of the matching ODporin percentile group from the no primary control. Background corrected OD values were log transformed (yielding ODCOX-I_T_, ODNDUFB8_T_ and ODporin_T_) to normalize the data.

The parameters (mean and standard deviation, SD) describing the distribution of ODporin_T_ in the control population (healthy and disease controls) was determined as described in the statistical methods, as well as parameters describing the linear relationship between ODCOX-I_T_ versus ODporin_T_ and ODNDUFB8_T_ versus ODporin_T_. Z-scores were determined for ODporin_T_ (porin__Z_), as well as Z-scores for NDUFB8 (NDUFB8__Z_) and COX-I (COX-I__Z_) based on the expected level of each according to the porin level.

Fibres were classified into groups according to porin Z-score: "very low" (porin__Z_ < −3SD), “low" (porin__Z_ between −3SD and −2SD), “normal" (porin__Z_ between −2SD and +2SD), "high" (porin__Z_ between +2SD and +3SD) and “very high" (porin__Z_ above +3SD). Fibres were similarly classified based on SD limits into groups of NDUFB8 and COX-I levels (normal if Z-scores > −3SD; intermediate(+) if Z-scores between −3SD and −4.5SD; intermediate(−) if Z-scores between −4.5SD to −6SD; deficient if Z-scores <−6 SD). The upper SD boundary of COX-I and NDUFB8 deficient groups were set as the highest NDUFB8__Z_ and COX-I__Z_ of red-appearing fibres on quadruple immunofluorescence (expressing porin but neither COX-I nor NDUFB8) from patients P11 and P17. These limits were confirmed and validated when immunofluorescence was performed on a serial section on a different day to assess reproducibility of the technique. The lower SD boundaries of COX-I and NDUFB8 positive groups were defined by the control group distribution.

### Statistical analyses

All statistical analyses were carried out using R 3.1.3^24^.

All data are presented as percentage (%) of the total number of myofibres, with n corresponding to the number of myofibres analysed.

#### Determination of porin, COX-I and NDUFB8 distribution in controls

The Box-Cox transformation^25^ identified the log transformation as optimal to achieve normality of the background corrected data. This was confirmed using both visual inspection and normality tests (Shapiro-Wilk^26^ and D’Agostino-Pearson’s omnibus test^27^).

The control group was created by simple random sampling of equal numbers of fibres from each of the control subjects, both healthy and disease controls. The control with the lowest number of fibres was determined and used to set the number of fibres randomly chosen in remaining controls. From the selected fibres, ODCOX-I_T_ versus ODporin_T_ and ODNDUFB8_T_ versus ODporin_T_ were plotted (Supplementary Fig. S2A and 2B). Linear regressions of ODCOX-I_T_ and ODNDUFB8_T_ (dependent variables) against ODporin_T_ (independent variable) were performed (Supplementary Fig. S2A and 2B), validated to ensure the residuals of the regression were normally distributed (Supplementary Fig. S2C and 2D) and the standard error of estimate was determined; this enabled the estimate of deviation of COX-I and NDUFB8 levels in each fibre from the predicted level according to the porin level.

#### Statistical validation of porin, COX-I, and NDUFB8 distribution in controls

The Z test for two population proportions was applied to assess the correlation of COX activity and COX-I abundance (Supplementary Table S2), and assess the reproducibility and reliability (Supplementary Table S3) of the quadruple immunofluorescence assay.

## Results

### Assessing the potential advantage of quadruple immunofluorescence over COX/SDH histochemistry

**Figure 1:**
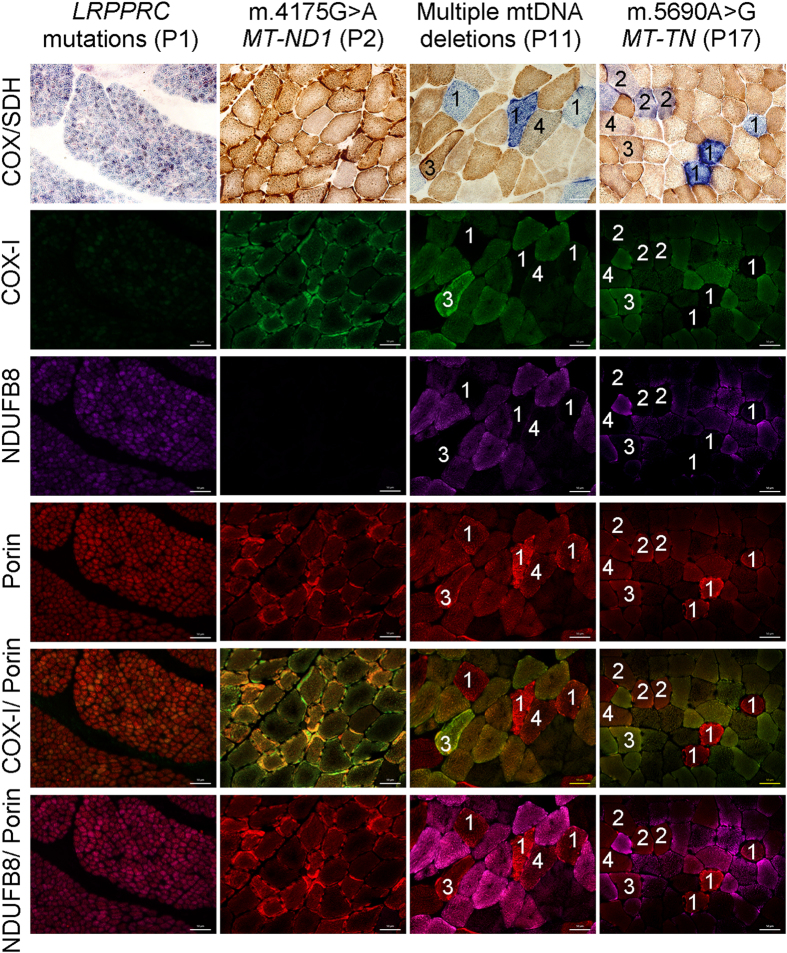
Comparison of COX/SDH histochemistry with complex I, IV and porin immunofluorescence. COX/SDH and quadruple immunofluorescence were performed in serial muscle sections from patients: P1, P2, P11 and P17. Fluorescent detection was used to visualise: complex IV subunit I (COX-I) - green (488 nm), complex I subunit (NDUFB8) - purple (647 nm) and porin (mitochondrial mass) - red (546 nm). P1 (*LRPPRC* mutations) shows widespread COX deficiency whereas P2 (m.4175G > A *MT-ND1* mutation) shows widespread NDUFB8 deficiency and preserved COX activity. Both P11 and P17 show mosaic COX deficiency. Selected muscle fibres demonstrate: **(1)** COX deficiency (COX-deficient fibres) with absent COX-I immunoreactivity, **(2)** decreased COX activity (COX-intermediate fibres) and decreased level of COX-I abundance, **(3)** normal COX activity (COX-positive fibres) and COX-I level but absent NDUFB8 immunoreactivity, **(4)** apparently normal COX activity and low level of COX-I immunoreactivity. All fibres highlighted show down-regulated levels of NDUFB8 abundance. P1 is a paediatric case and thus has smaller fibre size than the other cases. Scale bars measure 50 μm.

We first studied two patients with inherited genetic defects that lead to isolated deficiency of complex IV (P1) or complex I (P2), and two patients with mosaic COX deficiency (P11 and P17) (Table 1 and Fig. 1). Serial sections from these patients were assessed using COX/SDH and quadruple immunofluorescence. Based on the COX/SDH, three categories of myofibres were identified: COX-positive, COX-intermediate, and COX-deficient^28^.

The patient with *LRPPRC* mutations (P1) displayed uniform deficiency of COX-I consistent with the COX/SDH assay (Fig. 1). The patient with m.4175G > A *MT-ND1* (P2) had normal COX-I abundance and no observed deficiency following COX/SDH, but showed a marked defect in NDUFB8 abundance (Fig. 1). In both patients with mosaic deficiency of COX (P11 and P17), an agreement between COX activity and COX-I abundance was observed. Fibres with no discernible COX activity showed a complete absence of COX-I protein, those with lower COX activity had similarly decreased COX-I abundance, and fibres with enhanced COX activity showed the highest COX-I abundance. Interestingly, fibres exhibiting a subtle decrease in COX activity were barely distinguishable as abnormal based on COX/SDH (Fig. 1: fibres marked ″4″) although clearly abnormal in images of the merged COX-I/porin immunoreaction. All fibres highlighted in Fig. 1 showed decreased or absent NDUFB8.

### Correlation of COX/SDH histochemistry with quadruple immunofluorescence

**Figure 2:**
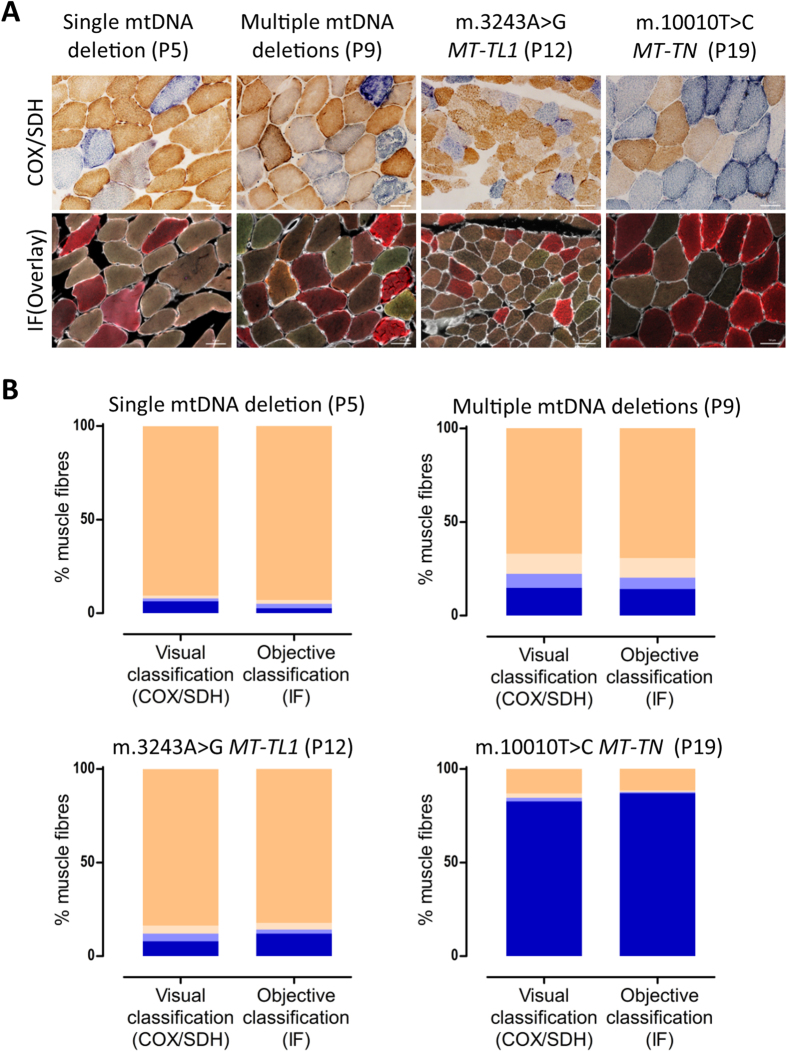
Correlation between COX activity and COX-I immunodetection. **(A)** COX/SDH histochemistry (top) and quadruple immunofluorescence (bottom, COX-I - green, NDUFB8 - purple, porin - red and laminin - white) were performed on serial muscle sections from patients P5, P9, P12 and P19. **(B)** Visual classification (COX/SDH) and objective classification (COX-I and porin immunodetection) results. Fibres were classified as COX (activity/protein abundance) positive (beige), intermediate(+) (light beige), intermediate(−) (light blue) or deficient (blue). Fibres counted (n = visual/immunodetection): P5 (n = 1103/841); P9: (n = 1395/1071); P12: (n = 1887/1740) and P19: (n = 956/769). Visually classified COX deficiency was overestimated in P5 by 3.6 percentage points, underestimated in P19 by 4.4 percentage points, underestimated in P12 by 4.1 percentage points, and consistent in P5, as compared to objective classification. Scale bars measure 50μm.

We investigated four different patients with mosaic COX deficiency evident with COX/SDH (P5, P9, P12, P19 (Table 1)). These patients were chosen because all muscle biopsies showed a distinction between COX-deficient and COX-intermediate fibres. Serial sections from these four patients were subjected to COX/SDH and quadruple immunofluourescence. Myofibres were visually classified based on COX/SDH; for improved classification the COX-intermediate group was subdivided into COX-intermediate(+) (Fig. 1, fibres ″4″ – grey cells) and COX-intermediate(−) (Fig. 1, fibres marked ″2″ – light blue/purple cells). The immunofluorescence was objectively classified based on COX-I and porin (Fig. 2A).

The difference between the objective COX-I classification and COX/SDH visual classification was always lower than 5 percentage points, but the difference between techniques was not systematic over- or under-estimation (Fig. 2B, Supplementary Table S2).

### Challenges of COX/SDH histochemistry in assessing degree of respiratory deficiency

**Figure 3:**
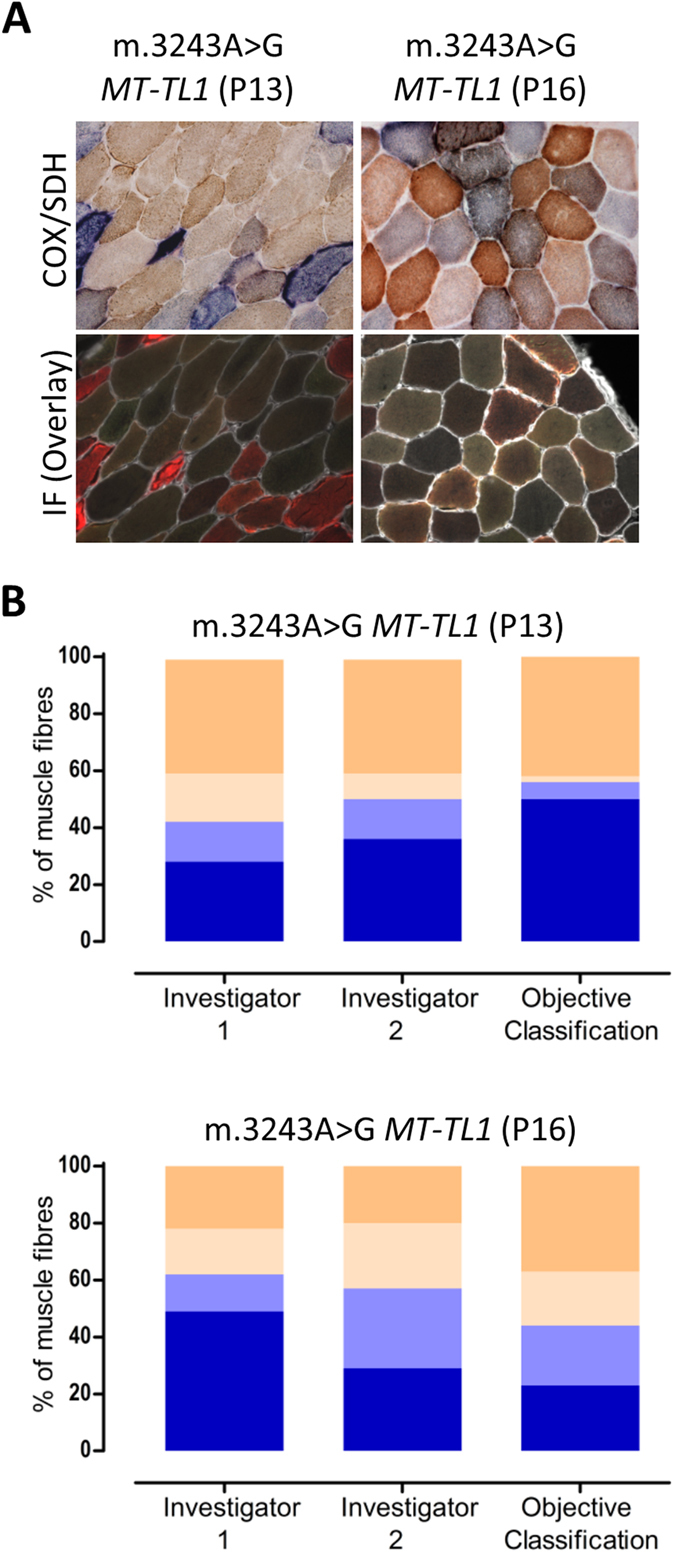
Inter- and intra-observer variability of visual classification. (**A**) COX/SDH (top) and quadruple immunofluorescence (bottom: COX-I - green, NDUFB8 - purple, porin - red and laminin - white) were performed in two serial muscle sections from patients P13 and P16. 100 myofibres from each patient were visually classified (COX/SDH) by two independent investigators, and objectively classified (COX-I and porin immunodetection). **(B)** Bar graphs show the percentage of COX-positive (beige), intermediate(+) (light beige), intermediate(−) (light blue) or deficient (blue) based on visual classification by investigator 1 and 2 (first two bars) and objective classification (last bar). In patient P13, both investigators identified the majority of COX-positive cells correctly (40% versus 42% using immunofluorescence) but differed in other categories. In patient P16, the investigators differed markedly in all categories. Importantly, both investigators demonstrated a high degree of internal inconsistency.

We investigated muscle biopsies from two patients which were difficult to visually assess, due to uneven labelling and an apparent continuous spectrum of COX activity: P13 and P16 (Table 1). Serial sections were assessed using COX/SDH and quadruple immunofluourescence (Fig. 3A). One hundred myofibres were selected and visually categorised by two researchers independently into one of the four categories: COX-positive, COX-intermediate(+), COX-intermediate(−) and COX-deficient. The same 100 fibres were objectively categorised based on COX-I and porin levels.

Analysis of both patients P13 and P16 revealed marked differences in fibre classification between the investigators, but importantly, this was also internally inconsistent; investigator 1 underestimated COX deficiency in patient P13 and overestimated it in patient P16, whereas the opposite was true for investigator 2 (Fig. 3B).

### Reproducibility and reliability of the quadruple immunofluorescent technique

We tested whether the immunofluorescent analysis of serial muscle sections from five patients (P1, P2, P11, P17 and P18) gave comparable results when sections are processed through the methodological protocol (quadruple immunofluorescence, imaging and IMARIS analysis) on two separate occasions (“day 1” and “day 2” 2 months apart) (Table 2). The results were highly consistent. The differences in the percentage of fibres constituting one of the four categories between the two time points varied between 0.1 and 9 percentage points.

**Figure 4:**
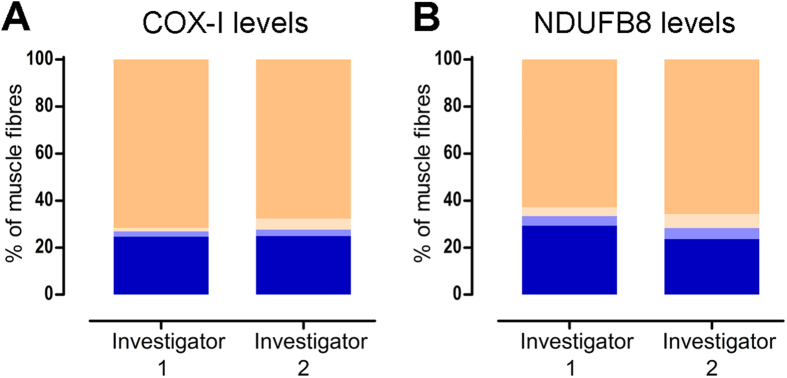
Inter-observer variability of quadruple immunofluorescence. Quadruple immunofluorescence was performed in a muscle section taken from P8 (multiple mtDNA deletions) and approximately the same area of the biopsy was assessed by investigator 1 (n = 528 fibres analysed) and investigator 2 (n = 470 fibres analysed). The assessment included imaging the selected area of the muscle section by each investigator on a separate occasion (15 days apart) and performing subsequent IMARIS analysis. Bar graphs show the percentage of fibres with normal (beige), intermediate(+) (light beige), intermediate(−) (light blue), and deficient (blue) levels of **(A)** COX-I and **(B)** NDUFB8, when assessed by investigators 1 and 2.

In order to evaluate the reliability of the method, we assessed whether the immunofluorescent analysis from the same tissue section (P8) gave comparable results when data are collected (imaging and IMARIS analysis) on two separate occasions by two investigators at a 15-day interval (Fig. 4). The results were almost identical for complex IV: the greatest difference of less than 4 percentage points was noted for COX-I positive and intermediate (+) groups (Fig. 4A, Supplementary Table S3). The results for complex I differed slightly more with the maximum discrepancy within the deficient fibres of 5.7 percentage points (Fig. 4B, Supplementary Table S3).

### Biochemical phenotype in genetically-defined mitochondrial myopathies

**Figure 5:**
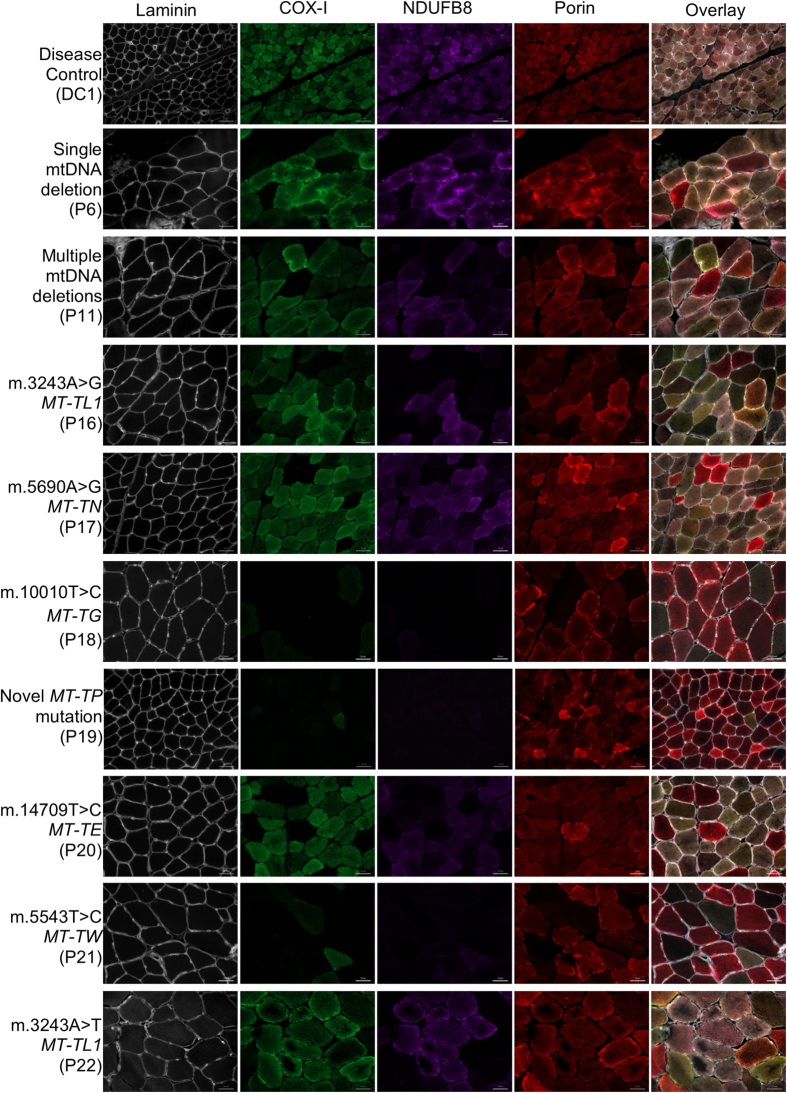
Representative images of complex I and IV abundance in skeletal muscle sections from control and disease cases using quadruple immunofluorescence. Fluorescent detection was used to visualise: COX-I - green, NDUFB8 - purple, porin - red and laminin – white. Representative images of respiratory-normal tissue (disease control, DC1) and single large-scale mtDNA deletion (P6), multiple mtDNA deletions (P11), m.3243A > G *MT-TL1* mutation (P16), m.5690A > G *MT-TN* mutation (P17), novel *MT-TP* mutation (P18), m.10010T > C *MT-TG* mutation (P19), m14709T > C *MT-TE* mutation (P20), m.5543T > C *MT-TW* mutation (P21) and m.3243A > T *MT-TL1* mutation (P22). Representative images of compound heterozygous *LRPPRC* mutations and m.4175G > A *MT-ND1* mutation are shown in Fig. 1. DC1 is a paediatric case and thus has smaller fibre size than the other cases. Scale bars measure 50 μm.

**Figure 6:**
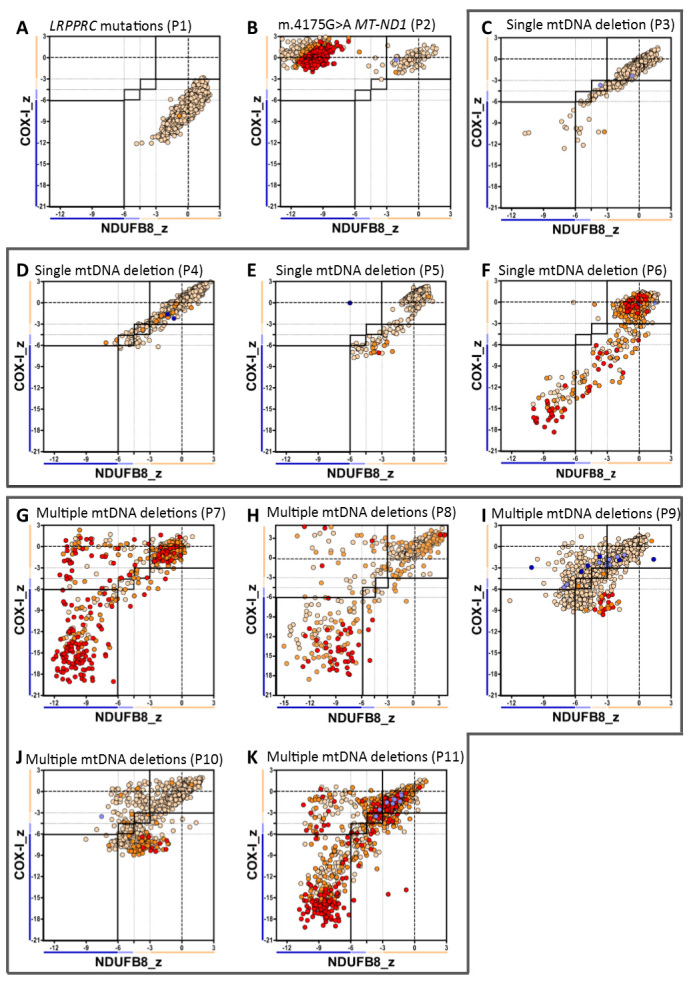
Mitochondrial respiratory chain expression profile linking complex I, complex IV and porin levels in patients with genetically-characterised mitochondrial disease. Plots show complex I and IV expression profile from patients with (**A**) isolated complex IV deficiency (compound heterozygous *LRPPRC* mutations, P1, n = 1258fibres analysed), (**B**) isolated complex I deficiency (m.4175G > A *MT-ND1* mutation, P2, n = 1062), (**C F**) single mtDNA deletion: (**C**) P3 (n = 1027), (**D**) P4 (n = 1228), (**E**) P5 (n = 841) and (**F**) P6 (n = 779), (**G K**) multiple mtDNA deletions: (**G**) P7 (n = 526), (**H**) P8 (n = 528), (**I**) P9 (n = 1071), (**K**) P10 (n = 1118) and (**K**) P11 (n = 2400), Each dot represents the measurement from an individual muscle fibre, colour coded according to its mitochondrial mass (very low: blue, low: light blue, normal: light orange, high: orange and very high: red). Thin black dashed lines indicate the SD limits for the classification of fibres, lines next to x and y axis indicate the levels of NDUFB8 and COX-I respectively (beige: normal, light beige: intermediate(+), light blue: intermediate(−) and blue: deficient). Bold dashed lines indicate the mean expression level of normal fibres.

Quadruple immunofluorescence was performed in twenty-two patients with genetically-determined mitochondrial disease (P1-P22) (Table 1). Representative cases are shown in Figs 1 and 5. We have developed a graphical way to present the data (Fig. 6) which shows NDUFB8 and COX-I protein abundance in conjunction with mitochondrial mass in individual myofibres. This clearly summarises the degree and type of OXPHOS deficiency.

#### Patients with known mutations affecting complex I or IV activities specifically

Patients P1 (*LRPPRC* mutations; Fig. 6A, Supplementary Table S4) and P2 (*MT-ND1* point mutation; Fig. 6B, Supplementary Table S4) showed isolated COX-I and NDUFB8 deficiencies respectively, in keeping with the biochemical defects assessed by spectrophotometric enzyme assay (Supplementary Table S5) and as predicted based on the known function of the mutated protein.

#### Single, large-scale mtDNA deletions

Patients harbouring single, large-scale mtDNA deletions (P3–P6, Fig. 6C–F) showed decreased abundance of both complex IV and complex I subunits with slightly more pronounced involvement of complex IV (Supplementary Table S4).

#### Multiple mtDNA deletions due to nuclear gene defects

Patients diagnosed with a nuclear-driven disorder leading to the accumulation of multiple mtDNA deletions in muscle (P7–P11, Fig. 6G–K) showed a greater proportion of fibres deficient in complex I than complex IV (Supplementary Table S4). All five patients presented a similar profile of biochemical deficiency: a discrete population of fibres that showed decreased NDUFB8 abundance without concomitant complex IV deficiency and a further, distinct population of myofibres in which subunits of both complex I and complex IV were equally down-regulated (Fig. 6G–K).

#### m.3243A>G MT-TL1 mutation

**Figure 7:**
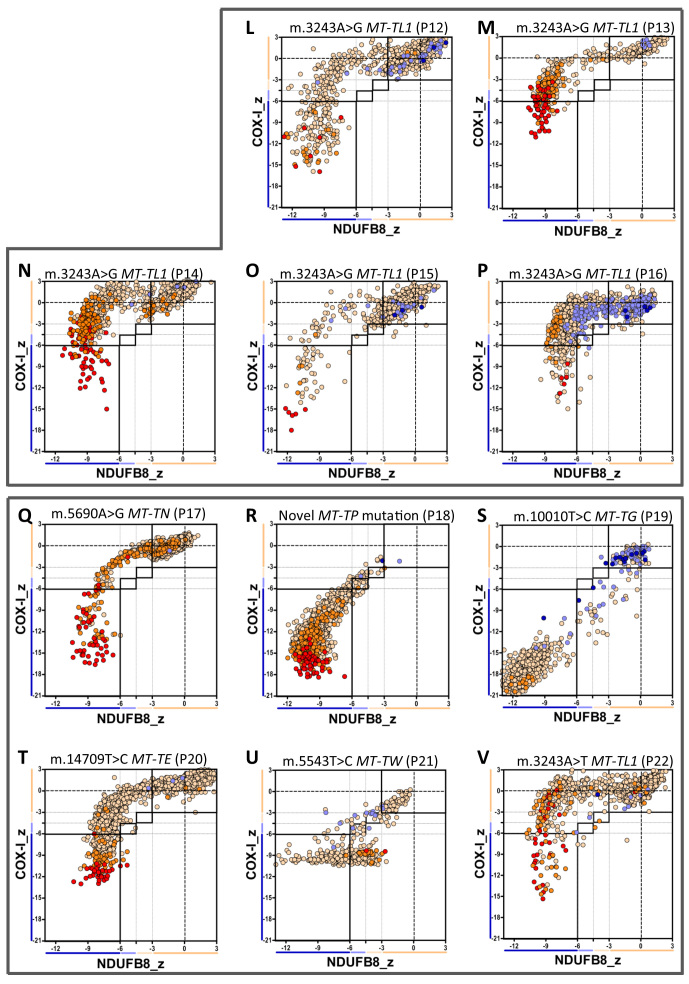
Continuation of figure 6. Plots show complex I and IV expression profile from patients with (**L-P**) m.3243A > G *MT-TL1* mutation: (L) P12 (n = 1328), (M) P13 (n = 499), (**N**) P14 (n = 1441), (**O**) P15 (n = 741) and (**P**) P16 (n = 918) and (**Q-V**) different mt-RNA mutations: (**Q**) m.5690A > G MT-TN mutation (P17, n = 1512), (**R**) novel MT-TP mutation (P18, n = 1012), (**S**) m.10010T > G MT-TG mutation (P19, n = 769), (**T**) m.14709T > C MT-TE mutation (P20, n = 1782), (**U**) m.5543T > C MT-TW mutation (P21, n = 447) and (**V**) m.3243A > T MT-TL1 mutations (P22, n = 1042).

The profile of deficiency in patients with this mutation (P12–P16, Fig. 7L–P) was similar, with much more severely affected complex I than complex IV (Supplementary Table S4).

#### Other mt-tRNA mutations

Patient P22, carrying the m.3243A > T *MT-TL1* mutation (fig.7V), had an OXPHOS deficiency similar to patients with m.3243A > G *MT-TL1* mutation (P12–P16, Fig. 7L–P). P17 (m.5690A > G *MT-TN* mutation, Fig. 7Q) and P20 (m.14709T > C *MT-TE* mutation, fig.7T) also showed more complex I than complex IV deficiency, but with more evidence of complex IV involvement than in the case of patients with m.3243A > G *MT-TL1* mutation (Supplementary Table S4). By contrast, P18 (Novel *MT-TP* mutation, unpublished, fig.7R), P19 (m.10010T > G *MT-TG* mutation, fig.7S) and P21 (m.5543T > C *MT-TW* mutation, fig.7U) demonstrated very high levels of deficiency.

## Discussion

OXPHOS deficiency can be difficult to quantify accurately. We have developed a sensitive quadruple immunofluorescent technique which enables accurate quantification of key OXPHOS protein abundance (complex I and complex IV) and a mitochondrial mass marker (porin) in individual myofibres, all within a 10 μm tissue section. Using laminin to define cell boundaries enables automatic quantification of myofibres^29^. We demonstrate that this technique is sensitive and reliable in detecting and quantifying focal OXPHOS deficiency in human muscle, and show that different mitochondrial genotypes – both mtDNA and nuclear DNA driven - exhibit distinctive signatures of mitochondrial dysfunction. We also demonstrate the precision and reproducibility of this technique, highlighting its potential not only as a diagnostic tool but also as a means to study mechanisms of disease, age-related changes, disease progression, and therapeutic interventions including exercise.

### Advantages of the new quadruple immunofluorescent assay

COX/SDH histochemistry is commonly used to assess OXPHOS function in individual cells and is extremely useful to screen for signs of mitochondrial dysfunction^7^. We have demonstrated that, in cases of obvious COX deficiency, the visual classification of fibres was comparable with the objective immunofluorescence. In these instances, we have shown that COX-I protein abundance correlated well with COX activity, validating the use of immunofluorescence to evaluate the mitochondrial biochemical profile. However, in challenging cases, visual classification was inconsistent and significantly differed between investigators.

Objective and informatics-based assessment allows greater accuracy and consistency in results^15^,^30^,^31^,^32^. We have demonstrated that our protocol overcomes the mis-estimation of mitochondrial dysfunction due to subjectivity, tissue-specific artefacts (such as uneven labelling) and limitations attributable to human error, through collecting data on two separate occasions by two investigators. We have demonstrated that this technique is reliable and reproducible by processing sections through the methodological protocol on two separate occasions. Additionally, the large numbers of experimental replicates conducted support the reproducibility of this technique, since all the controls demonstrated consistent and comparable levels of protein abundance, and importantly, almost all patients exhibited at least a small population of fibres with Z-scores centred appropriately around the mean NDUFB8 and COX-I levels observed in the controls.

Previous studies have elegantly explored the involvement of multiple OXPHOS complexes using immunohistochemistry for the diagnosis and understanding of mitochondrial pathology^33^,^34^,^35^,^36^,^37^, but there have been limited studies to quantify the deficiency^28^,^35^,^38^. Our method quantitates mitochondrial protein abundance using a single muscle section, avoiding potential confounds of section thickness variance and changing biochemical profiles along myofibres^28^,^39^,^40^,^41^.

Though our data demonstrates the close association of COX-I abundance and COX enzymatic activity, there is no histochemical assay for complex I able to accurately measure activity in single muscle fibres, making validation for this complex difficult. However, it has been shown that mutations in many subunits of the OXPHOS complexes lead to assembly defects or affect complex stability, leading to degradation of the unintegrated proteins^42^,^43^. Importantly, NDUFB8 has been shown to be essential to the functional enzymatic activity of Complex I^23^, and thus reduced protein abundance can reliably be interpreted as lowered enzymatic activity.

### Biochemical phenotype of different mitochondrial genetic defects

We have shown that specific genotypes present similar patterns of mitochondrial deficiency, consistent with the known biochemical defect. For example, patients harbouring compound heterozygous *LRPPRC* mutations showed isolated COX deficiency^44^,^45^,^46^, whilst the patient carrying the m.4175G > A *MT-ND1* mutation showed isolated complex I deficiency^47^,^48^,^49^,^50^.

Patients with single, large-scale mtDNA deletions show a simultaneous loss of both complex I and complex IV, consistent with both the lack of mtDNA encoded structural subunits of OXPHOS complexes and the loss of several mt-tRNAs. Though all single deletion patients demonstrated simultaneous loss of both complexes, there is variation in the linear direction or curve of decline between patients, suggesting differential rates of COX-I decline in comparison to NDUFB8, possible related to the site and size of the deletions^51^.

Patients with nuclear gene mutations leading to a disturbance of mtDNA maintenance demonstrated a similar pattern of enzyme deficiency, independent of the underlying molecular genetic defect. The nature of the biochemical defect in these muscle biopsies is different from that seen in biopsies from patients with single, large-scale mtDNA deletions, compatible with previous reports^52^. The presence of fibres with complex I deficiency is consistent with reports of the loss of complex I structural genes in the deleted molecules^53^,^54^, although further molecular studies are necessary to correlate the biochemical and molecular phenotype in individual myofibres^55^.

Mutations in mtDNA-encoded tRNA genes are associated with impaired mtDNA-encoded protein translation, leading to multiple OXPHOS defects. Interestingly, patients with the common m.3243A > G *MT-TL1* mutation demonstrate a pattern of deficiency (the distinctive (rotated) L shape seen repeatedly in Fig. 7L–P) suggesting that complex IV is only affected after complex I deficiency is already established, consistent with previous observations^56^. This pattern also indicates that the deficiency is smoothly graduated from normal to deficient levels of both complexes, contrasting with the polarised pattern observed in the *MT-ND1* mutation. As patients with this mutation demonstrate highly variable clinical manifestations, it would be interesting to see if this technique can reveal a correlation between clinical and biochemical phenotypes^57^. The patient carrying a transversion mutation at this site, m.3243A > T *MT-TL1*, showed the same pattern of deficiency, despite being reported to present a higher deleterious effect on translation^58^. However, there are several potential confounders to any interpretation of the severity of the defect, not least heteroplasmy.

Rarer mt-tRNA point mutations revealed other distinct patterns of deficiency. Though the mutations studied in *MT-TN* and *MT-TE* show the same pattern of deficiency as seen in the *MT-TL1* mutations, those in *MT-TG* and *MT-TP* instead suggested simultaneous decline in both complex I and complex IV protein abundance. A third distinctive pattern is seen in the *MT-TW* mutation; although severe deficiency is observed in both complexes, the deficiency in complex IV appears to plateau at a level much higher than observed in other mt-tRNA point mutations. Additionally, though the majority of fibres with complex IV deficiency demonstrated that COX-I abundance has declined to a specific basal level, a wide range of NDUFB8 deficiency is observed across the same fibres. This suggests that the relative rate of decline in complex I and complex IV protein levels varies considerably from fibre to fibre for this mutation, an effect not observed in other mt-tRNA point mutations.

For all the mtDNA mutations, future investigations are required to correlate the observed biochemical profile with heteroplasmy levels, and similarly, for mt-tRNA mutations specifically, the correlation of the biochemical profile with the relative amounts of the cognate amino acid within each of the mtDNA-encoded complex I and IV proteins.

### Technical methodology

A major and recurrent problem when performing immunofluorescence is signal from non-specific binding of secondary antibodies. Several research groups have developed strategies to mitigate these effects, usually by manually setting an intensity threshold to identify positive cells^59^,^60^. Nevertheless this threshold is subjective. For our analyses, the non-specific signal was objectively measured on the NPC and subtracted from the intensities measured on the quadruple immunofluorescence. We found that the secondary antibody (IgG1 biotinylated) coupled with NDUFB8 showed a higher affinity in fibres with high porin abundance, evident from higher intensity fluorescence on the NPC. We identified that this off-target binding was associated with the anti-IgG1secondary antibody and not the fluorophore (Alexa Fluor 647), since exchanging the secondary antibody fluorophores resulted in the increased NPC signal switching to the alternate fluorophore (Alexa Fluor 546).

To compensate for this effect and ensure the NDUFB8 values represent true signal, the ODNDUFB8 signal were corrected according to mitochondrial mass. This approach was validated by the fact that, without correction, ragged red fibres (which have dense mitochondrial mass and hence porin, but low abundance of COX-I and NDUFB8^61^) demonstrated constant basal levels of COX-I signal but ODNDUFB8 signal proportional to the ODporin. With correction the ODNDUFB8 in ragged red fibres was at a constant basal level consistent with the COX-I abundance and no increasing trend with ODporin was observed for either OXPHOS protein.

A supporting tool written in R^24^ to automate the analysis is available on request.

## Conclusion

We have developed an objective, reliable and high-throughput quantification technique that allows assessment of complex I and complex IV protein abundance relative to mitochondrial mass in individual fibres from patients with mitochondrial myopathies using a single (10 μm) muscle section. This work has yielded important insights into the nature of the biochemical defects in patients with mitochondrial dysfunction. We believe this new technique has important implications for the diagnosis and treatment of mitochondrial diseases and other diseases with mitochondrial involvement, as well as investigating the molecular mechanisms underlying these conditions.

## Additional Information

**How to cite this article**: Rocha, M. C. *et al.* A novel immunofluorescent assay to investigate oxidative phosphorylation deficiency in mitochondrial myopathy: understanding mechanisms and improving diagnosis. *Sci. Rep.* **5**, 15037; doi: 10.1038/srep15037 (2015).

## Supplementary Material

Supplementary Information

## Figures and Tables

**Table 1:** Clinical information from patients included in this study.

Subjects	Gender	Age	Clinical information	Genetic defect
Patients with known mutations affecting complex I and IV specifically				
P1	Male	1y	Encephalopathy, seizures, hypotonia, developmental delay, central apnoeas	Compound heterozygous *LRPPRC* mutations (manuscript in preparation)
P2^a^	Male	20y	Exercise-induced muscle weakness and lactic acidosis	m.4175G > A (p.Trp290*) *MTND1* mutation
Single, large-scale mtDNA deletion				
P3	Female	22y	CPEO	single, large-scale mtDNA deletion
P4	Female	29y	CPEO and bilateral ptosis	single, large-scale mtDNA deletion
P5	Female	39y	CPEO	single, large-scale mtDNA deletion
P6	Female	74y	CPEO	single, large-scale mtDNA deletion
Autosomal disorders of mtDNA maintenance				
P7^b^	Male	36y	Severe PEO, ptosis, proximal muscle weakness, facial weakness, scapulae winging, low BMI, hypogonadism and osteoporosis.	multiple mtDNA deletions; autosomal recessive p.(Thr144Ile) and p.(Gly273Ser) *RRM2B* mutations
P8^c^	Female	43y	Severe PEO, asymmetrical ptosis, proximal and distal muscle weakness, ataxia, SNHL, facial weakness, low BMI, leukoencephalopathy and depression	multiple mtDNA deletions; autosomal recessive p.(Arg186Gly) and p.(Thr218Ile) *RRM2B* mutations
P9	Male	47y	CPEO and bilateral ptosis	multiple mtDNA deletions; autosomal dominant p.(Asp104Gly) *SLC25A4* mutation
P10	Female	54y	CPEO, ptosis, muscle weakness	autosomal dominant p.(Gln458His) *PEO1* mutation
P11	Male	60y	CPEO and bilateral ptosis	multiple mtDNA deletions; nuclear basis unresolved
m.3243A > G *MT-TL1* mutation				
P12	Female	25y	Exercise intolerance, ptosis	m.3243A > G *MT-TL1* mutation
P13	Female	40y	Exercise intolerance and mild deafness	m.3243A > G *MT-TL1* mutation
P14	Female	42y	Epilepsy, bilateral sensorineural hearing loss, diabetes, gastrointestinal complications	m.3243A > G *MT-TL1* mutation
P15	Female	47y	Modest exercise intolerance	m.3243A > G *MT-TL1* mutation
P16	Male	53y	CPEO	m.3243A > G *MT-TL1* mutation
Other mt-RNA mutations				
P17^d^	Female	13y	CPEO, ptosis, proximal myopathy	m.5690A > G *MT-TN* mutation
P18	Female	18y	Seizures, deafness, retinopathy	Novel m.16021_16022detCT *MT-TP* mutation (unpublished)
P19	Male	33y	Mitochondrial myopathy	m.10010T > C *MT-TG* mutation
P20	Female	35y	Mild weakness	m.14709T > C *MT-TE* mutation
P21	Male	63y	Pure exercise intolerance, prominent exertional dyspnea	m.5543T > C *MT-TW* mutation
P22	Male	76y	Muscle pain and weakness	m.3243A > T *MT-TL1* mutation
Healthy control				
HC1	Female	20y	Biopsy obtained during orthopaedic surgery.	n.a.
Disease controls				
DC1	Male	4y	Hypotonia and fatigue	n.a.
DC2	Male	18y	Leukodystrophy and raised creatine kinase	n.a.
DC3	Female	20y	Tremor and spasticity	n.a.

Key: age = age when biopsied; y = years old; mtDNA = mitochondrial DNA; PEO = progressive external ophthalmoplegia; CPOE = chronic CPEO; BMI = body mass index; ^a,b,c,d^published cases.

^a^Gorman *et al.* (2015) in press.

^b^P19 and.

^c^P20 in Pitceathly *et al.* (2012).

^d^P3 in Blakely *et al.* (2013).

**Table 2:** Quantification of COX-I and NDUFB8 deficiency in two serial skeletal muscle sections from 5 patients.

Patients		COX-I				NDUFB8				
		Pos	Int(+)	Int(−)	Neg	Pos	Int(+)	Int(−)	Neg	n
P1 (*LRPPRC* mutations)	Day 1	0.2%	3.8%	15.3%	80.7%	99.8%	0.2%	0.0%	0.0%	424
	Day 2	0.2%	4.5%	17.3%	78.0%	99.6%	0.3%	0.1%	0.0%	1258
	E (D1–D2)	0.1%	−0.8%	−2.0%	2.7%	0.2%	−0.1%	−0.1%	−	
	95% CI (D1–D2)	−0.4%, 0.6%	−2.9%, 1.4%	−6.0%, 2.0%	−1.7%, 7.1%	−0.4%, 0.7%	−0.6%, 0.5%	−0.2%, 0.1%	−	
P2 (m.4175G > A *MT-ND1*)	Day 1	99.9%	0.1%	0.0%	0.0%	9.6%	0.4%	0.2%	89.7%	823
	Day 2	100.0%	0.0%	0.0%	0.0%	11.9%	0.3%	0.1%	87.8%	932
	E (D1–D2)	−0.1%	0.1%	−	−	−2.3%	0.2%	0.1%	2.0%	
	95% CI (D1–D2)	−0.3%, 0.1%	−0.1%, 0.3%	−	−	−5.0%, 0.5%	−0.4%, 0.7%	−0.2%, 0.5%	−0.8%, 4.8%	
P11(Multiple mtDNA deletions)	Day 1	72.8%	5.3%	3.2%	18.7%	63.4%	12.4%	4.7%	19.6%	1589
	Day 2	71.2%	5.2%	2.7%	21.0%	54.4%	19.0%	4.1%	22.5%	2400
	E (D1–D2)	1.7%	0.1%	0.5%	−2.3%	9.0%	−6.6%	0.6%	−2.9%	
	95% CI (D1–D2)	−1.2%, 4.5%	−1.3%, 1.5%	−0.6%, 1.6%	−4.8%, 0.3%	5.9%, 12.1%	−8.9%, −4.4%	−0.7%, 1.9%	−5.5%, 0.3%	
P17 (m.5690A>G *MT-TN*)	Day 1	91.5%	1.0%	1.2%	6.2%	79.6%	7.1%	3.3%	10.0%	1362
	Day 2	91.5%	1.1%	1.2%	6.2%	81.2%	5.9%	3.6%	9.3%	1512
	E (D1–D2)	0.0%	−0.1%	0.1%	0.0%	−1.6%	1.2%	−0.3%	0.7%	
	95% CI (D1–D2)	−2.0%, 2.1%	−0.9%, −0.7%	−0.8%, 0.9%	−1.8%, 1.8%	−4.5%, 1.3%	−0.6%, 3.0%	−1.6%, 1.1%	−1.5%, 2.8%	
P18 (Novel *MT-TP* mutation)	Day 1	0.5%	1.1%	2.5%	95.9%	0.4%	0.5%	4.4%	94.7%	1012
	Day 2	0.4%	0.9%	1.6%	97.0%	0.2%	0.4%	2.2%	97.2%	1689
	E (D1–D2)	0.1%	0.1%	0.9%	−1.1%	0.2%	0.1%	2.3%	−2.6%	
	95% CI (D1–D2)	−0.5%, 0.6%	−0.7%, 0.9%	−0.3%, 2.0%	−2.6%, 0.4%	−0.2%, 0.7%	−0.5%, 0.6%	0.8%, 3.2%	−4.1%, −1.0%	

Key: E (D1–D2) = estimate for difference between Day 1 and Day 2; 95% CI (D1-D2) = 95% confidence interval for the difference between Day 1 and Day 2; n = number of fibres quantified; Pos = positive, Int(+) = intermediate positive, Int(−) = intermediate negative, Neg = negative.
