# Supplementary material for: A novel immunofluorescent assay to investigate oxidative phosphorylation deficiency in mitochondrial myopathy: understanding mechanisms and improving diagnosis
Source: Sci Rep. 2015 Oct 15;5:15037. doi: 10.1038/srep15037 (PMC4606788; doi:10.1038/srep15037)
Supplement: Supplementary Information [file srep15037-s1.pdf]

A novel immunofluorescent assay to investigate oxidative phosphorylation deficiency in mitochondrial myopathy: understanding mechanisms and improving diagnosis

Mariana C. Rocha[1,2#], John P. Grady[1#], Anne Grünewald[2], Amy Vincent[2], Philip F. Dobson[1] Robert W. Taylor[2], Doug M. Turnbull[1,2], Karolina A. Rygiel[1,2*]

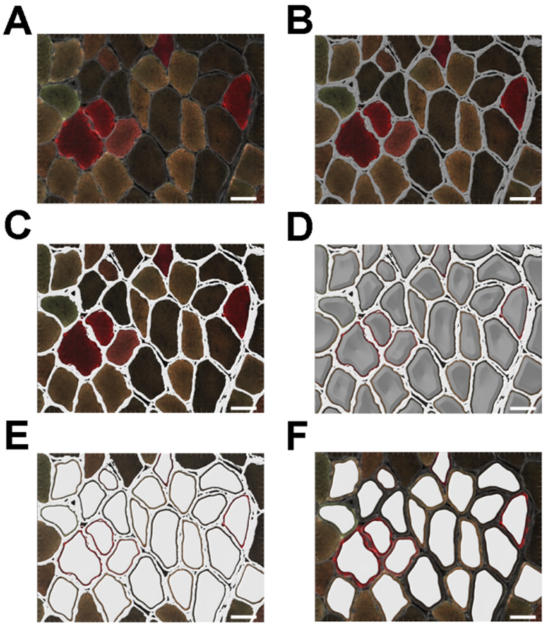

Supplementary Fig. S1. Representative print-screen images showing the main steps of segmentation using IMARIS software. (A) Images were opened in IMARIS as 16-bit czi files. **(B)** A first surface was created over the 405 nm channel in order to fully cover laminin labelling. **(C)** This surface was then used to create a laminin mask. **(D)** A second surface was created over the masked 405 nm channel thereby filling the area of muscle fibres. **(E)** Using "edit" tool bar unwanted areas, such as background, were removed. **(F)** The mean intensity of all four channels (405 nm, 488 nm, 546 nm, 647 nm) was measured automatically for selected individual muscle fibres. Scale bars measure 40µm.

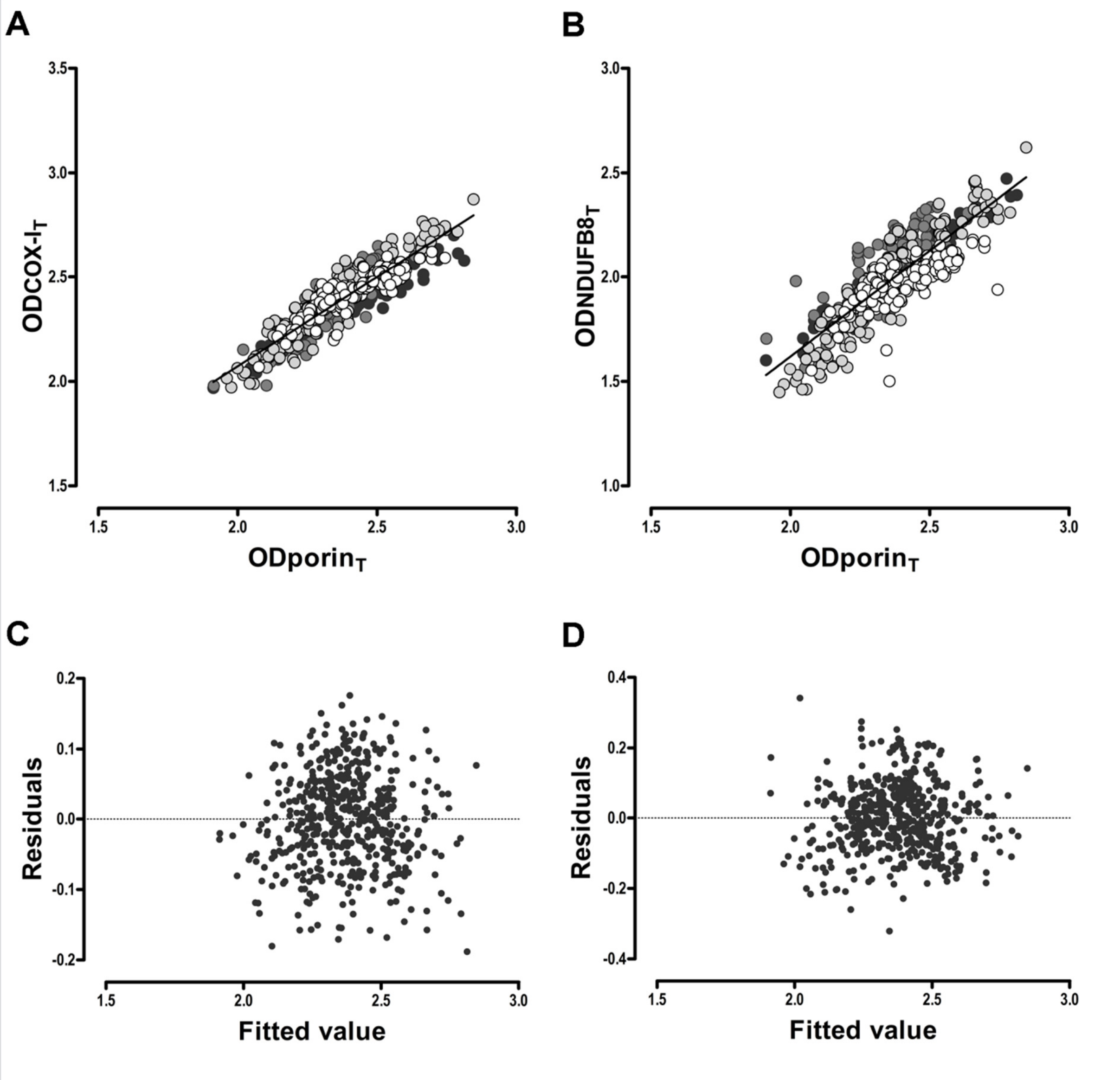

Supplementary Fig. 2. Creation of the control group and establishment of ODCOX-I$_T$ and ODNDUFB8$_T$ linear regressions. 128 fibres from each healthy and disease controls were randomly selected. **(A)** ODCOX-I$_T$ versus ODporin$_T$ and **(B)** ODNDUFB8$_T$ versus ODporin$_T$ were plotted together (Black circles: HC, dark grey circles: DC1, light grey circles: DC2, white circles: DC3) and used to perform the linear regressions of ODCOX-I$_T$ and ODNDUFB8$_T$. The residual plots from **(C)** ODCOX-I$_T$ linear regression and **(D)** ODNDUFB8$_T$ linear regression showed a normal distribution validating the linear regression.

Supplementary Table S1. Primary and secondary antibodies used for immunofluorescence

Antibodies	Host	Dilution	Company (Product number)
**Primary antibodies**			
Laminin (IgG)	Rabbit	1:50	Sigma-Aldrich (L9393)
COX-I (IgG2a)	Mouse	1:100	Abcam (Ab14705)
Porin (IgG2b)	Mouse	1:100	Abcam (Ab14734)
NDUFB8 (IgG1)	Mouse	1:100	Abcam (Ab110242)
**Secondary antibodies**			
Anti-rabbit Alexa Fluor 405 nm	Goat	1:200	Life Technologies (A31556)
Anti-IgG2a Alexa Fluor 488 nm	Goat	1:200	Life Technologies (A21131)
Anti-IgG2b Alexa Fluor 546 nm	Goat	1:200	Life Technologies (A21143)
Anti-IgG1 biotin	Goat	1:200	Jackson IR Lab (115-065-205)
Streptavidin Alexa Fluor 647 nm	Goat	1:100	Life Technologies (S31556)

Key: Jackson IR Lab = Jackson ImmunoResearch Laboratories

Supplementary Table S2. Correlation of COX activity and COX-I expression

Patients		Complex IV				
		Pos	Int(+)	Int(-)	Neg	n
**P5 (Single mtDNA deletion)**	**VC**	90.5%	1.5%	1.6%	6.3%	1103
	**OC**	92.9%	2.1%	2.2%	2.7%	841
	**E(VC-OC)**	-2.4%	-0.6%	-0.6%	3.6%	
	**p-value**	0.06	0.34	0.33	>0.05	
**P9 (Multiple mtDNA deletions)**	**VC**	67.0%	10.8%	7.5%	14.8%	1395
	**OC**	69.3%	10.5%	6.0%	14.2%	1071
	**E(VC-OC)**	-2.3%	0.3%	1.5%	0.6%	
	**p-value**	0.21	0.81	0.14	0.69	
**P12 (m.3243A>G *MT-TL1*)**	**VC**	83.6%	4.3%	4.1%	8.0%	1887
	**OC**	82.2%	3.6%	2.2%	12.0%	1740
	**E(VC-OC)**	1.4%	0.8%	1.8%	-4.1%	
	**p-value**	0.25	0.23	>0.05	>0.05	
**P19 (m.10010T>C *MT-TG*)**	**VC**	13.1%	2.3%	2.0%	82.6%	956
	**OC**	11.6%	0.8%	0.7%	87.0%	769
	**E(VC-OC)**	1.5%	1.5%	1.3%	-4.4%	
	**p-value**	0.34	>0.05	>0.05	>0.05	

Key: VC = visual classification based on COX/SDH histochemistry; OC = objective classification based on quantitative immunofluorescence; E(VC-OC) = estimate for difference between VC and OC; Z test for 2 population proportions.

Supplementary Table S3. Inter-observer variability of quadruple immunofluorescence.

Patient P8		COX-I / NDUFB8 levels<br>Pos	Int(+)	Int(-)	Neg	n
COX-I levels	Invest. 1	67.7%	4.7%	2.8%	24.9%	470
	Invest. 2	71.6%	1.5%	2.3%	24.6%	528
	E(I1-I2)	-3.9%	3.2%	0.5%	0.3%	
	p-value	0.18	>0.05	0.62	0.92	
NDUFB8 levels	Invest. 1	65.7%	6.0%	4.7%	23.6%	470
	Invest. 2	62.9%	3.8%	4.0%	29.4%	528
	E(I1-I2)	2.9%	2.2%	0.7%	-5.7%	
	p-value	0.35	0.11	0.59	0.04	

Key: Invest. 1 = investigator 1; Invest. 2 = investigator 2; E(I1-I2) = estimate for difference between investigator 1 and investigator 2; Z test for 2 population proportions.

Supplementary Table S4. Quantification of both COX-I and NDUFB8 deficiency and porin levels from all patients with mitochondrial disease.

Patient	COX-I					NDUFB8					Porin					
	Pos	Int(+)	Int(-)	Neg	Abnormal	Pos	Int(+)	Int(-)	Neg	Abnormal	V.High	High	Normal	Low	V.Low	n
**Patients with known mutations affecting complex I and IV specifically**																
**P1 (*LRPPRC* mutations)**	0.2%	4.5%	17.3%	78.0%	99.8%	99.6%	0.3%	0.1%	0.0%	0.4%			100%	<		1258
**P2 (m.4175G>A *MT-ND1*)**	99.9%	0.1%	0.0%	0.0%	0.1%	11.9%	0.3%	0.1%	87.8%	88.1%	34%	20%	45%	<		1062
**Single, large-scale mtDNA deletion**																
**P3**	92.6%	4.3%	2.7%	0.4%	7.4%	93.1%	4.9%	1.8%	0.3%	6.9%			98%	1%	<	1027
**P4**	92.7%	4.4%	1.3%	1.6%	7.3%	96.3%	1.9%	1.1%	0.7%	3.7%		<	99%	<		1228
**P5**	92.6%	2.3%	2.3%	2.9%	7.4%	95.6%	2.6%	1.7%	0.1%	4.4%	<	1%	99%		<	841
**P6**	81.5%	2.1%	2.3%	14.1%	18.5%	88.4%	2.2%	2.2%	7.2%	11.6%	8%	25%	67%	<		779
**Autosomal disorders of mtDNA maintenance (multiple mtDNA deletions)**																
**P7**	60.1%	3.4%	3.8%	32.7%	39.9%	49.2%	3.4%	4.4%	43.0%	50.8%	32%	35%	34%			526
**P8**	71.6%	1.5%	2.3%	24.6%	28.4%	62.9%	3.8%	4.0%	29.4%	37.1%	9%	28%	63%			528
**P9**	69.0%	10.2%	6.3%	14.5%	31.0%	62.0%	13.6%	14.4%	10.0%	38.0%	1%	1%	96%	1%	1%	1071
**P10**	72.4%	5.1%	3.4%	19.1%	27.6%	70.6%	14.8%	11.7%	2.9%	29.4%	1%	5%	94%		<	1118
**P11**	71.2%	5.2%	2.7%	21.0%	28.8%	54.4%	19.0%	4.1%	22.5%	45.6%	7%	21%	71%	2%	<	2400

**m.3243A>G *MT-TL1* mutation**																
**P12**	83.7%	2.5%	1.8%	12.0%	16.3%	73.7%	3.1%	1.5%	21.7%	26.3%	1%	2%	95%	3%	<	1328
**P13**	69.1%	11.0%	9.4%	10.4%	30.9%	48.5%	3.4%	2.8%	45.3%	51.5%	9%	15%	75%	1%		499
**P14**	90.6%	4.4%	1.8%	3.2%	9.4%	64.6%	3.3%	2.4%	29.6%	35.4%	4%	8%	87%	<		1441
**P15**	89.1%	3.0%	1.3%	6.6%	10.9%	78.8%	7.4%	1.9%	11.9%	21.2%	1%	1%	96%	3%	<	741
**P16**	67.0%	16.7%	7.0%	9.4%	33%	30.6%	5.7%	11.8%	52.0%	69.4%	1%	3%	79%	17%	1%	918
**Other mt-RNA mutations**																
**P17 (m.5690A>G *MT-TN*)**	91.5%	1.1%	1.2%	6.2%	8.5%	81.2%	5.9%	3.6%	9.3%	18.8%	1%	2%	97%	1%	<	1512
**P18 (Novel *MT-TP* mutation)**	0.5%	1.1%	2.5%	95.9%	99.5%	0.4%	0.5%	4.4%	94.7%	99.6%	1%	6%	92%	<		1012
**P19 (m.10010T>C *MT-TG*)**	11.6%	0.8%	0.7%	87.0%	88.4%	13.5%	2.2%	1.3%	83.0%	86.5%		2%	90%	6%	2%	769
**P20 (m.14709T>C *MT-TE*)**	74.1%	5.7%	4.8%	15.5%	25.9%	58.0%	3.3%	6.3%	32.5%	42%	2%	4%	94%	<		1782
**P21 (m.5543T>C *MT-TW*)**	13.6%	5.1%	3.6%	77.6%	86.4%	14.3%	15.2%	26.4%	44.1%	85.7%	<	4%	93%	3%		447
**P22 (m.3243A>T *MT-TL1*)**	89.6%	2.9%	1.8%	5.7%	10.4%	68.0%	3.0%	3.8%	25.1%	32.0%	3%	5%	91%	1%	<	1042

Key: n = number of fibres quantified, abnormal = abnormal fibres, either int(+), int(-) or neg, < = proportion of fibres lower than 0.5%

Supplementary Table S5. Activity of the respiratory chain complexes measured in muscle homogenates from P1 and P2.

Patients	Complex I/CS	Complex II/CS	Complex III/CS	Complex IV/CS
**P1 (*LRPPRC* mutations)**	0.062	0.096	0.472	0.242
**P2 (m.4175G>A *MT-ND1*)**	0.001	0.070	0.634	1.216
**Control (n=25)**	0.104±0.036	0.145±0.047	0.554±0.345	1.124±0.511

Enzyme activities are expressed as nmols NADH oxidised.min^{-1}.unit citrate synthase (CS)$^{-1}$ for complex I, nmols DCPIP reduced.min^{-1}.unit citrate synthase^{-1} for complex II and the apparent first-order rate constant (K).sec^{-1}.unit citrate synthase^{-1} for complexes III and IV (x 10^3). Control values are shown as mean±standard deviation. DCPIP = 2,6-dichlorophenolindophenol
